# Health professionals’ perception of patient safety culture in a university hospital in São Paulo: A cross-sectional study applying the Hospital Survey on Patient Safety Culture

**DOI:** 10.1590/1516-3180.2018.0430140319

**Published:** 2019-07-22

**Authors:** Julia Hiromi Hori Okuyama, Taís Freire Galvão, Marcia Terezinha Lonardoni Crozatti, Marcus Tolentino Silva

**Affiliations:** I MSc. Pharmacist at Hospital São Paulo, Hospital Universitário, Universidade Federal de São Paulo (HU-UNIFESP), and Doctoral Student, Postgraduate Program on Pharmaceutical Sciences, Universidade de Sorocaba (UNISO), Sorocaba (SP), Brazil.; II MSc, PhD. Pharmacist and Professor, Faculdade de Ciências Farmacêuticas (FCF), Universidade Estadual de Campinas (UNICAMP), Campinas (SP), Brazil.; III MSc, PhD. Pharmacist and Professor, Pharmaceutical Sciences Course, Universidade Federal de São Paulo (UNIFESP), Diadema (SP), Brazil.; IV MSc, PhD. Pharmacist and Professor, School of Medicine, Universidade Federal do Amazonas (UFAM), Manaus (AM), and Professor, Postgraduate Program on Pharmaceutical Sciences, Universidade de Sorocaba (UNISO), Sorocaba (SP), Brazil.

**Keywords:** Patient safety, Organizational culture, Surveys and questionnaires, Hospitals, Safety management

## Abstract

**BACKGROUND:**

Patient safety culture is part of the organizational profile of healthcare institutions and is associated with better quality of care.

**OBJECTIVE:**

To assess patient safety culture in a university hospital.

**DESIGN AND SETTING:**

Hospital-based cross-sectional study conducted in a public university hospital in São Paulo, Brazil, between September and December 2015.

**METHODS:**

We randomly selected 68 sectors of the hospital, to include up to 5 employees from each sector, regardless of length of experience. We used the validated Brazilian version of the Hospital Survey on Patient Safety Culture (HSOPS) via an electronic interface. We calculated the percentage of positive responses for each dimension of the HSOPS and explored the differences in age, experience, occupation and educational level of respondents using the chi-square test.

**RESULTS:**

Out of 324 invited respondents, 314 (97%) accepted the invitation and were surveyed. The sample presented predominance of women (72%), nursing staff (45%) and employees with less than six years’ experience at the hospital (60%). Nine out of the 12 dimensions showed percentages of positive responses below 50%. The worst results related to “nonpunitive response to errors” (16%). A better safety culture was observed among more experienced staff, nurses and employees with a lower educational level. In the previous year, no events were reported by 65% of the participants.

**CONCLUSIONS:**

The patient safety culture presented weaknesses and most of professionals had not reported any event in the previous year. A policy for improvement and cyclical assessment is needed to ensure safe care.

## INTRODUCTION

New technologies associated with increased knowledge regarding healthcare have changed the operation of hospital environments, such that they have become more complex.^1^ Simpler treatments have been replaced by sets of procedures requiring continual training and supervision of healthcare professionals. The inherent risks of these more complex systems, combined with insufficient investment, professional overload, communication failures and inadequate supervision in hospital environments, can result in occurrences of adverse events.^2^

Since the 1999 publication of the report “To err is human: Building a safer health system”^2^ by the Institute of Medicine, healthcare organizations have increased their focus on issues relating to patient safety.^3^ Data on mortality due to adverse events have become available, especially in the United States, in addition to data on the social costs caused by irreversible harm to users and their families.^2^

An update of these data has shown that medical errors are the third leading cause of death in the United States.^4^ In settings with lower resources, this risk may be higher. The incidence of adverse events in Brazil has been estimated to be 7.6% for hospitalized patients, and it has been shown that 66.7% could be avoided. These findings are similar to those from studies in other countries.^5^

Healthcare institutions are viewed as organizations that are based on a culture founded on values, attitudes, skills and standards of individual and group behavior, which define quality in healthcare. Patient safety culture is part of the organizational habits of hospital institutions,^1^ and an assessment of patient safety culture makes it possible to obtain knowledge on the factors that are involved in the professionals’ routine and their perceptions, along with the strengths and weaknesses of the culture of patient safety. Such assessments also make it possible to identify the sectors and processes that generate risks.^6^ Knowing the weaknesses in patient safety culture makes it feasible to establish interventions and improvements in the quality of care for users, thus changing the professionals’ behavior.^7^

To assess patient safety culture, surveys with validated questionnaires are widely used.^8^ The Hospital Survey on Patient Safety Culture (HSOPS) and the Safety Attitudes Questionnaire have been widely cited in research that aimed to assess patient safety culture in hospital settings worldwide.^6^ The HSOPS, developed by the United States Agency for Healthcare Research and Quality (AHRQ) in 2004,^9^ proposes 12 dimensions to assess patient safety culture from the professionals’ perspective in a hospital setting (Table 1). This instrument has been translated and validated for use in several languages,^10^^,^^11^^,^^12^ and in 2012, the Brazilian version was made available for use.^13^ In 2013, the Brazilian government issued regulations on patient safety actions, including identification, reporting and system improvement.^14^^,^^15^

**Table 1;:** Dimensions of the Hospital Survey on Patient Safety Culture: numbers of items and what is assessed^9^

Dimensions of patient safety culture relating to the work area or unit		
1. Teamwork within units	4 items	Assessment of teamwork, considering support by supervisors and managers, open communication about mistakes and continuous improvement of errors within teams
2. Supervisor/manager expectations and actions that promote patient safety	4 items	
3. Organizational learning and continuous improvement	3 items	
4. Communication openness	3 items	
5. Feedback and communication about error	3 items	
6. Staffing	4 items	
7. Nonpunitive response to errors	3 items	
Dimensions that explore aspects of safety culture in a hospital		
8. Management support for patient safety	3 items	Assessment of hospital management support for patient safety and cooperation between units to maintain the quality of and information on patient care
9. Teamwork across units	4 items	
10. Handoffs and transitions (i.e. handovers)	4 items	
Dimensions of outcome measurements		
11. Overall perceptions of patient safety	4 items	Assessment of the existence of procedures to avoid occurrences of errors and make rectifications before errors impact the patient
12. Frequency of events reported	3 items	

## OBJECTIVE

Few studies have reported on use of the HSOPS in Brazil to characterize the level of patient safety culture in Brazilian hospitals. In this scenario, studies that estimate patient safety culture are necessary. The objective of this study was to assess perceptions of patient safety culture in a university hospital.

## METHODS

### Study design and context

This was a hospital-based cross-sectional study in which the HSOPS was used to assess patient safety culture from the professionals’ perspective. It was conducted from September to December 2015 at Hospital São Paulo, the university hospital of Universidade Federal de São Paulo, located in the city of São Paulo, the largest city in Brazil. This hospital provides high-complexity care in all medical specialties and has more than 700 beds. The primary outcome was the percentage of positive responses for each dimension of the HSOPS.

### Participants

All professionals who directly or indirectly were attending patients in the hospital, regardless of their length of experience at the institution, were eligible for participation in this study. Trainees, interns, dismissed employees and outsourced workers (cleaning, security and food service employees) were not eligible, because not including them would improve the homogeneity of the sample.

### Sample size and sampling process

To calculate the sample size, we considered the population of approximately 5,000 employees at the hospital. We made a conservative estimate for the frequency of positive responses regarding the presence of patient safety culture (“strongly agree/agree” or “most of the time/always”) of 50%. In the dimensions of the HSOPS, a precision rate of 7%, a value of 1.5 for the sampling effect and a possible loss rate of 10% were used. These parameters resulted in a need to survey a minimum of 312 professionals.

We randomly selected 60 primary and 20 secondary sectors out of the 106 sectors of the main building of the hospital and invited up to five employees who were present at the time of the visit to each sector, to be interviewed.

### Data collection

The instrument used for data collection was the Brazilian version of the HSOPS.^13^ The survey is composed of 42 items grouped into 12 dimensions that measure different aspects of patient safety culture, including personal data relating to the professional and data on the unit and the hospital (Table 1). The HSOPS makes it possible to measure the beliefs, skills and behaviors involved in the safety culture of the organization from hospital staff perspectives.

Each dimension is composed of three to four items that are constructed in a positive or negative manner (Table 1). For each item, the respondent may choose a score on a five-point Likert scale with the response options of strongly agree, agree, neither agree nor disagree, disagree and strongly disagree, or response options of never, rarely, sometimes, most of the time and always, in relation to frequency.^9^ Two other items assess individual assessments of patient safety: the “patient safety grade”, with response options of excellent, very good, acceptable, poor and failing, and the “number of events reported”, with response options of no events reported, 1 to 2 events reported, 3 to 5 events reported, 6 to 10 events reported, 11 to 20 events reported and 21 or more events reported.

After reversing the sentences that were negatively worded, we calculated the percentage of positive responses regarding the presence of patient safety culture in each dimension by dividing the number of positive responses (“strongly agree/agree” or “most of the time/always”) by the total number of responses (positive, neutral and negative) in the dimension. A percentage of positive responses above 75% was considered strong, and a percentage below 50% showed that there were issues that needed improvement. For items with reverse wording and that had a negative connotation, disagreement indicated a positive response. Thus, to calculate the percentage of positive responses among the answers, we needed to consider the strongly disagree/disagree or never/rarely responses.

In the process of pretesting the survey, we modified the Portuguese-language wording of three items (A5 in the “staffing” dimension, C1 in the dimension of “feedback and communication about error” and G1 in the dimension of “number of events reported”), in accordance with previous recommendations, to improve comprehensibility (Table 1).^16^ The research group that suggested this wording has, furthermore, validated a new version of the HSOPS in an electronic interface.^17^

The survey was developed using a suite of tools for field data called KoBo Toolbox (www.kobotoolbox.org, Cambridge, MA, USA) and was administered in the workplace. Notices invited hospital staff to participate in the study and, after agreeing to do so and signing an informed consent form, staff members completed the survey using tablet electronic devices (Samsung Galaxy Tab 3). The device recorded the data online or offline and, after connecting to the internet, the surveys were automatically uploaded to the online platform.

Two trained survey administrators performed the data collection: a pharmacy undergraduate student and a pharmacist.

### Statistical methods

The negatively worded items were reverse-coded to calculate the percentage of positive responses for each dimension. The answers were recoded as follows: strongly disagree, disagree, neither agree nor disagree, always, most of the time and sometimes were assigned a score of 0; while agree, strongly agree, never and rarely were assigned a score of 1, in accordance with the HSOPS manual.^9^

The proportion of positive responses for each dimension was stratified according to respondent age, length of employment at the hospital (in years: less than 1; 1 to 5; 6 to 10; 11 to 20; or 21 or more), profession (doctor, nurse or other professional) and educational level (completion of high school, undergraduate level or postgraduate level). The differences were tested using the chi-square test and were considered significant if P < 0.05.

To assess the internal consistency of the survey, we calculated Cronbach’s alpha for each dimension and item of the Brazilian version of the HSOPS. The calculations on the data were done using Stata 14.2.

### Ethical issues

The present study was approved by the hospital’s research ethics committee, under the number CAAE 48415315.3.0000.5505. All subjects signed an informed consent form.

## RESULTS

We invited 324 employees from 68 sectors of the hospital to participate. A total of 314 professionals (97%) accepted the invitation and were included, while 10 (3%) refused to participate.

Most participants were women (72%); 41% had undergraduate and postgraduate educational levels. The majority had direct contact with patients (80%), 45% were nursing staff (nurses, nursing technicians and nursing assistants) and 60% had been working at the hospital for less than six years. As shown in Table 2, different professionals participated in the survey.

**Table 2.** Characteristics of the respondents (n = 314)

Characteristics	Sample (n)	Frequency (%)
Age (years)		
18-34	145	46.2
35-44	84	26.8
45-70	85	27.1
Gender		
Female	226	72.0
Male	88	28.0
Educational level		
Elementary and high school	104	33.1
Undergraduate level at college/university	82	26.1
Postgraduate level	128	40.8
Length of employment at the hospital (years)		
Less than 1	59	18.8
1 to 5	94	29.9
6 to 10	25	8.0
11 to 20	98	31.2
21 or more	38	12.1
Professional experience in the work area/unit (years)		
Less than 1	67	21.3
1 to 5	123	39.2
6 to 10	29	9.2
11 to 20	69	22.0
21 or more	26	8.3
Working hours per week		
Less than 40	126	40.1
40 to 59	140	44.6
60 or more	48	15.3
Staff position		
Physician	53	16.9
Nurse	142	45.2
Other professional (pharmacists, therapists, etc.)	35	11.2
Technicians (laboratory, radiology)	19	6.1
Management/secretary	26	8.3
Other	39	12.4
Direct patient interaction		
Yes	252	80.3
No	62	19.8
Professional experience in the same position or specialty (years)		
Less than 1	37	11.8
1 to 5	100	31.9
6 to 10	25	8.0
11 to 20	104	33.1
21 or more	48	15.3

Nine out of the 12 dimensions showed positive response rates below 50% (Table 3). The dimension of “nonpunitive response to errors” had the worst result (16%). A total of 65% of the participants indicated that they had reported no events in the past 12 months. The internal consistency was adequate for eight dimensions and the other four showed lower consistency (Cronbach’s alpha < 0.6).

**Table 3.** Percentage of positive responses according to dimension (n = 314)

Dimensions	%	95% CI	Cronbach’s alpha
Supervisor/manager expectations and actions promoting patient safety	53.0	49.2-56.8	0.75
Organizational learning and continuous improvement	51.5	47.9-55.1	0.56
Teamwork within units	51.0	47.5-54.5	0.62
Frequency of events reported	43.8	39.2-48.5	0.89
Communication openness	40.0	36.1-43.9	0.68
Feedback and communication about error	35.7	31.8-39.6	0.70
Overall perceptions of patient safety	34.7	31.7-37.8	0.48
Staffing	28.0	25.2-30.8	0.53
Handoffs and transitions (i.e. handovers)	26.8	23.6-29.9	0.66
Teamwork across units	24.8	22.6-27.1	0.61
Management support for patient safety	23.0	19.4-26.7	0.76
Nonpunitive response to errors	15.6	13.2-18.1	0.37

CI = confidence interval.

Greater age and length of work experience were associated with higher perceptions of patient safety culture in the dimensions of “supervisor/manager expectations and actions promoting patient safety”, “organizational learning and continuous improvement”, “frequency of events reported”, “feedback and communication about error”, “staffing” and “management support for patient safety”. On the other hand, the dimension of “nonpunitive response to errors” was only associated with age (Table 4). The dimension of “frequency of events reported” was significantly different according to professional category (higher perception among nurses than among other professionals and physicians) and educational level (lower perception among employees with higher education). The dimension of “management support for patient safety” was also inversely proportional to educational level.

**Table 4.** Frequency of positive responses in the dimensions of patient safety culture, stratified according to subgroups (n = 314)

Variables	Dimensions of patient safety culture											
	1	2	3	4	5	6	7	8	9	10	11	12
Age group (years)												
18-24	44.6	51.8	44.0	16.7	25.9	25.0	34.5	22.6	17.9	20.5	29.5	15.5
25-34	49.8	52.6	47.9	17.4	29.5	30.5	34.5	39.0	23.7	25.0	25.6	12.3
35-54	56.8	53.6	59.1	20.6	40.5	40.1	49.2	48.8	26.8	31.0	25.3	14.7
55-70	59.7	66.1	64.5	46.2	48.4	51.6	44.1	68.8	33.1	39.5	33.1	29.0
P-value	0.132	0.135	0.012	< 0.001	0.003	0.001	0.083	< 0.001	0.095	0.020	0.571	0.009
Length of experience (years)												
< 1	52.1	58.1	45.2	21.5	31.8	32.2	37.3	32.8	24.2	22.9	30.9	13.0
1-5	49.7	50.5	50.4	25.2	31.6	32.6	36.2	42.9	23.4	25.0	26.3	15.6
6-10	43.0	48.0	50.7	8.0	35.0	33.3	34.7	52.0	17.0	34.0	25.0	14.7
11-15	57.1	50.7	55.4	20.3	35.8	35.1	45.5	43.2	29.4	28.7	26.7	13.5
16-20	57.3	62.5	55.6	20.8	36.5	44.4	48.6	58.3	26.0	28.1	25.0	18.1
≥ 21	55.3	67.1	68.4	43.0	52.6	55.3	51.8	61.4	31.6	45.4	30.9	27.2
P-value	0.304	0.037	0.027	< 0.001	0.023	0.003	0.059	< 0.001	0.227	0.008	0.864	0.076
Staff position												
Physician	55.7	62.3	48.4	18.2	31.6	30.8	34.6	31.4	25.0	25.9	26.9	15.7
Nurse	55.5	53.0	56.3	25.1	37.3	38.7	47.4	51.2	28.7	31.3	33.1	17.6
Other	51.2	52.7	52.1	23.4	35.3	37.5	39.3	46.3	25.3	28.3	25.0	14.2
P-value	0.771	0.299	0.535	0.474	0.692	0.454	0.175	0.013	0.804	0.698	0.413	0.804
Educational level												
High school or less	54.3	59.9	60.3	36.9	39.9	46.8	43.3	58.7	26.7	32.5	34.6	18.3
Undergraduate	50.9	51.8	49.6	17.5	33.2	31.7	37.4	38.2	25.0	24.7	23.8	15.4
Postgraduate	52.3	52.3	49.7	17.2	34.2	32.0	42.2	38.8	25.2	28.7	24.4	14.8
P-value	0.890	0.435	0.219	0.001	0.566	0.041	0.666	0.004	0.956	0.475	0.158	0.772

Note: Dimensions: (1): Teamwork within units; (2): Supervisor/manager expectations and actions promoting patient safety; (3): Organizational learning and continuous improvement; (4): Management support for patient safety; (5): Overall perceptions of patient safety; (6): Feedback and communication about error; (7): Communication openness; (8): Frequency of events reported; (9): Teamwork across units; (10): Staffing; (11): Handoffs and transitions (i.e. handovers); (12): Nonpunitive response to errors.

## DISCUSSION

Patient safety culture in this hospital was fragile, considering that 9 of the 12 dimensions of HSOPS were rated at below 50%. Two-thirds of the respondents did not report any events in the last 12 months, thus indicating that potential safety problems may be going unrecognized and are not being addressed properly. The low rate of positive responses for the dimension of “nonpunitive response to errors” has also been found in other studies,^18^^,^^19^^,^^20^ and this may also explain the behavior of not reporting events.

The dimensions with higher levels of positive responses, i.e. “supervisor/manager expectations and actions promoting patient safety”, “organizational learning and continuous improvement” and “teamwork within units”, did not represent strengths in patient safety culture, since they fell below 75%.^9^ Within their work units, professionals may seek to carry out their activities in a team with supervised support and to look for improvements to patient safety.^21^ Teamwork is a critical point and is important because it relies on collaboration and mutual respect.^21^ Such values lead to opportunities to adopt improvement programs. Investigations conducted by different researchers have found similar results.^18^^,^^22^^,^^23^

A study that applied the HSOPS to 26 hospitals in Iran^20^ observed that there was better perception in the dimension of “organizational learning and continuous improvement”. In teaching hospitals, professionals are willing to improve their understanding and knowledge. It has been observed that in the dimension of “organizational learning and continuous improvement”, the percentage of positive responses improves as the amount of work experience increases.^24^

The “staffing” dimension needs improvement, which may be an effect caused by a situation of an insufficient number of professionals with heavy workloads. This imbalance increases the risk relating to the assistance provided.^10^ In units that perform activities under unfavorable conditions, professionals feel that the level of support that they can count on to carry out their tasks safely when they are confronted by a high volume of responsibilities is lower.^25^

The number of working hours can also be related to the results, since tiredness decreases attention and increases the incidence of errors.^26^ A number of factors affect the safety and quality of patient care, such as the organization of nursing units, structure, communication, stress and workload.^27^ A better distribution of professionals and appropriate working hours are paramount for improving healthcare quality.

The dimensions of “communication openness” and “feedback and communication about error” indicated that there was a need for to improve priorities. Ineffective communication increases the occurrence of adverse events.^24^ As observed in other studies, failure in communication is directly related to worsening of quality of care.^28^^,^^29^ Hospitals in which there is a channel for free communication between supervisors and employees to exchange suggestions, questions and feedback on improvements in patient safety tend to have better scores for quality and motivation, with regard to learning from errors.^18^

Professionals with greater experience had a better perception of safety culture. Usually, such professionals have more responsibility or occupy leadership positions within their teams. This may positively influence their perception of patient safety, as observed in a study conducted in Finland that compared the perceptions of managers and registered nurses.^30^ The experience of a professional can positively influence the results, as shown in a Palestinian study in which the number of adverse events reported increased with a professional’s length of experience.^19^ The participants in the present study were mostly composed of early-career professionals, which may explain the low rate of errors reported. More events were reported by nurses than by the medical team, which is similar to what was seen in a study conducted in the United States.^31^

Given that contextual limitations may have influenced the present results, we need to highlight that an employee strike had ended just before the time of data collection and that budget cuts occurred during the survey period. Despite the difficulties faced by these professionals, a good acceptance rate was obtained for the survey. The participants were a diverse group of professionals who were either directly or indirectly involved with patient care. Examining the hospital as a whole improves the representativeness of the results.^32^ We also chose to approach employees in person instead of via remote strategies, which are more prone to give rise to a less diverse sample population and a lower response rate. The institution surveyed here is a university hospital and its staff include a wide variety of professionals for the purposes of undergraduate education, residency and specialization. These data may suggest that high turnover exists,^33^^,^^34^ and this may have been related to the low perception of safety among these professionals.

The reliability of the HSOPS version used in the present study was fair. Changes that had been made to improve comprehensibility^16^ resulted in better consistency in the “staffing” dimension, such that it improved from 0.20 in the first Brazilian validation of the HSOPS^13^ to 0.53 in the present study. A new validation of the HSOPS that featured better wording of these questions was performed and published after our survey was conducted and had high instrument reliability.^17^

The negative results found in the present study may be viewed as demotivating with regard to patient safety in the hospital. Measuring safety culture is the first step towards identifying the priorities that need to be addressed if a change in patient safety is to be achieved. In Brazil, the regulations in this field are still evolving, and greater investment in patient safety strategies is required.^14^^,^^15^ In addition to ameliorating assistance, improvement of patient safety culture in university hospitals enriches undergraduate and postgraduate education.

## CONCLUSION

Patient safety culture in this Brazilian hospital was shown to be fragile, and improvement is necessary in order to ensure safe care. Implementation of enhancement measures and further assessment of patient safety culture should be a cyclical process to drive effective changes in patient safety forward.

## References

[1] Sousa P, Mendes W (2014). Segurança do paciente: criando organizações de saúde seguras.

[2] Kohn LT, Corrigan JM, Donaldson MS, Institute of Medicine (US) Committee on Quality of Health Care in America (2000). To Err Is Human: Building a Safer Health System.

[3] WHO (2004). World Alliance for Patient Safety: forward programme 2005.

[4] Makary MA, Daniel M (2016). Medical error-the third leading cause of death in the US. BMJ.

[5] Mendes W, Martins M, Rozenfeld S, Travassos C (2009). The assessment of adverse events in hospitals in Brazil. Int J Qual Health Care.

[6] Pumar-Méndez MJ, Attree M, Wakefield A (2014). Methodological aspects in the assessment of safety culture in the hospital setting: a review of the literature. Nurse Educ Today.

[7] Wakefield JG, McLaws ML, Whitby M, Patton L (2010). Patient safety culture: factors that influence clinician involvement in patient safety behaviours. Qual Saf Health Care.

[8] Colla JB, Bracken AC, Kinney LM, Weeks WB (2005). Measuring patient safety climate: a review of surveys. Qual Saf Health Care.

[9] Sorra JS, Nieva VF (2004). Hospital Survey on Patient Safety Culture. (Prepared by Westat, under Contract No. 290-96-0004). AHRQ Publication no. 04-0041.

[10] Chen IC, Li HH (2010). Measuring patient safety culture in Taiwan using the Hospital Survey on Patient Safety Culture (HSOPSC). BMC Health Serv Res.

[11] Hellings J, Schrooten W, Klazinga NS, Vleugels A (2010). Improving patient safety culture. Int J Health Care Qual Assur.

[12] Gama ZA, Oliveira AC, Hernández PJ (2013). Cultura de seguridad del paciente y factores asociados en una red de hospitales públicos españoles [Patient safety culture and related factors in a network of Spanish public hospitals]. Cad Saude Publica.

[13] Reis CT, Laguardia J, Vasconcelos AG, Martins M (2016). Reliability and validity of the Brazilian version of the Hospital Survey on Patient Safety Culture (HSOPSC): a pilot study. Cad Saúde Pública.

[14] Brasil. Ministério da Saúde (2013). Portaria nº 529, de 1º de abril de 2013. Institui o Programa Nacional de Segurança do Paciente (PNSP).

[15] Brasil. Ministério da Saúde. Agência Nacional de Vigilância Sanitária (2013). Resolução da Diretoria Colegiada - RDC nº 36, de 25 de julho de 2013. Institui ações para a segurança do paciente em serviços de saúde e dá outras providências.

[16] Gama ZA, Batista AM, Silva IG, Souza RM, Freitas MR (2013). [Cross-cultural adaptation of the Brazilian version of the Hospital Survey on Patient Safety Culture: opportunities for improvement]. Cad Saude Publica.

[17] Andrade LEL, Melo LOM, Silva IGD (2017). Adaptation and validation of the Hospital Survey on Patient Safety Culture in an electronic Brazilian version. Epidemiol Serv Saude.

[18] Alahmadi HA (2010). Assessment of patient safety culture in Saudi Arabian hospitals. Qual Saf Health Care.

[19] Hamdan M, Saleem AA (2013). Assessment of patient safety culture in Palestinian public hospitals. Int J Qual Health Care.

[20] Raeissi P, Reisi N, Nasiripour AA (2018). Assessment of Patient Safety Culture in Iranian Academic Hospitals: Strengths and Weaknesses. J Patient Saf.

[21] Sammer CE, Lykens K, Singh KP, Mains DA, Lackan NA (2010). What is patient safety culture? A review of the literature. J Nurs Scholarsh.

[22] Agnew C, Flin R, Mearns K (2013). Patient safety climate and worker safety behaviours in acute hospitals in Scotland. J Safety Res.

[23] Fujita S, Seto K, Ito S (2013). The characteristics of patient safety culture in Japan, Taiwan and the United States. BMC Health Serv Res.

[24] Aboshaiqah AE, Baker OG (2013). Assessment of nurses’ perceptions of patient safety culture in a Saudi Arabia hospital. J Nurs Care Qual.

[25] Vlayen A, Hellings J, Claes N, Abdou EA, Schrooten W (2015). Measuring safety culture in Belgian psychiatric hospitals: validation of the Dutch and French translations of the hospital survey on patient safety culture. J Psychiatr Pract.

[26] Wu Y, Fujita S, Seto K (2013). The impact of nurse working hours on patient safety culture: a cross-national survey including Japan, the United States and Chinese Taiwan using the Hospital Survey on Patient Safety Culture. BMC Health Serv Res.

[27] Khater WA, Akhu-Zaheya LM, Al-Mahasneh SI, Khater R (2015). Nurses’ perceptions of patient safety culture in Jordanian hospitals. Int Nurs Rev.

[28] Belyansky I, Martin TR, Prabhu AS (2011). Poor resident-attending intraoperative communication may compromise patient safety. J Surg Res.

[29] Kilner E, Sheppard LA (2010). The role of teamwork and communication in the emergency department: a systematic review. Int Emerg Nurs.

[30] Turunen H, Partanen P, Kvist T, Miettinen M, Vehviläinen-Julkunen K (2013). Patient safety culture in acute care: a web-based survey of nurse managers’ and registered nurses’ views in four Finnish hospitals. Int J Nurs Pract.

[31] Campbell EG, Singer S, Kitch BT, Iezzoni LI, Meyer GS (2010). Patient safety climate in hospitals: act locally on variation across units. Jt Comm J Qual Patient Saf.

[32] Loney PL, Chambers LW, Bennett KJ, Roberts JG, Stratford PW (1998). Critical appraisal of the health research literature: prevalence or incidence of a health problem. Chronic Dis Can.

[33] Shortell SM, Zimmerman JE, Rousseau DM (1994). The performance of intensive care units: does good management make a difference?. Medical Care.

[34] Vogus TJ, Cooil B, Sitterding M, Everett LQ (2014). Safety organizing, emotional exhaustion, and turnover in hospital nursing units. Medical Care.

